# Alignment of Epidemiology Practice and Academic Competencies through Effective Collaboration

**DOI:** 10.3389/fpubh.2016.00068

**Published:** 2016-04-25

**Authors:** Kimberly R. Glenn, Paula R. Masters, Megan A. Quinn

**Affiliations:** ^1^Office of Healthcare Statistics, Division of Policy, Planning and Assessment, Tennessee Department of Health, Nashville, TN, USA; ^2^Department of Student Services, College of Public Health, East Tennessee State University, Johnson City, TN, USA; ^3^Department of Biostatistics and Epidemiology, College of Public Health, East Tennessee State University, Johnson City, TN, USA

**Keywords:** epidemiology, workforce capacity, modules, government, academics, collaboration

## Abstract

**Background:**

Online learning has recently garnered increased attention as technology use in the classroom grows. However, most of the published approaches regarding this topic in postgraduate education centers on clinical environments. Models of partnerships between applied public health agencies and academic centers to produce mutually beneficial online learning opportunities for graduate-level public health courses have not been explored in the literature.

**Methods:**

East Tennessee State University (ETSU) and the Tennessee Department of Health (TDH) partnered to build three online, asynchronous epidemiology modules for an interdisciplinary audience of graduate students. The goals of the modules were to (1) introduce students to a public health issue, (2) provide students with hands-on learning about data and information available through TDH, and (3) allow students to connect theory to practice by having them create a product for use by TDH. TDH created topic-specific modules that would be used within the infectious disease, chronic disease, and cancer epidemiology courses, and piloted during the 2015–2016 academic term.

**Results:**

Conference calls between the two institutions occurred in the spring and the summer of 2015. Two of the three epidemiology modules were presented to ETSU staff for critique and edits at an in-person meeting during the summer. The methods of delivery for each section within a module varied from recorded webinar format to self-guided instruction. One module utilized available learning tools provided by the Centers of Disease Control and Prevention, while the other module was constructed entirely using TDH data. Both modules included various exercises and assignments to be conducted in class and as homework and concluded with the student being asked to construct a learning product as a final project. The ETSU–TDH team decided that this learning product would be provided back to TDH for possible future use.

**Discussion:**

The innovative partnership between a state government agency and an academic institution has demonstrated the need for such collaborations in public health. Understanding how applied public health practice would utilize what is learned in the classroom and preparing students for real-world application may be the missing link between theory and practice.

## Introduction and Background

### Site

The Tennessee *L*ong-Distance *I*nternet *F*acilitated *E*ducational *P*rogram for *A*pplied *T*raining in *H*ealth (Tennessee LIFEPATH) was a workforce development partnership aimed at public health employees in the state of Tennessee. Tennessee, like all states, is vulnerable to deficits in the human resources infrastructure of its public health system, particularly as the aging workforce retires, as it responds to ongoing budget crises, and as it recognizes that its public health workforce is under-prepared for leadership. Tennessee LIFEPATH created a partnership that provides training to Tennessee’s public health workforce. Tennessee LIFEPATH was housed at the East Tennessee State University (ETSU) College of Public Health, a Council on Education for Public Health (CEPH)-accredited Association of Schools and Programs of Public Health (ASPPH) member school, and serves as the Local Performance Site for the Region IV Public Health Training Center at Emory University funded by the Health Resources and Services Administration (HRSA). LIFEPATH had strong partnerships with the Tennessee Department of Health (TDH), Tennessee Public Health Association (TPHA), and the National Association of City and County Health Officials (NACCHO). LIFEPATH’s vision was to be a vital collaboration that will provide multiple entry points for public health workers to obtain public health education and training designed to improve their skills, experiences, and competencies.

Long-Distance Internet Facilitated Educational Program for Applied Training in Health provided a wide range of programs to assure that Tennessee’s public health workforce has the knowledge, skills, and training to meet Tennessee’s present and forthcoming health challenges. Tennessee LIFEPATH serves all of Tennessee. The public health professionals in these areas were afforded the opportunity to access LIFEPATH training programs to become a more competent workforce and in turn impact the overall health of all its residents. Trainings were offered through a variety of avenues such as online continuing education trainings, public health lectures series, comprehensive, region-specific courses and workshops, and conferences. Tennessee LIFEPATH touched a very diverse population with its training. Tennessee has both metropolitan areas and widely dispersed people living in rural areas, especially in Appalachia. This diverse population has little resources by way of training for its public health workers. The Center provides opportunities statewide that previously were not available. Since its inception, the Center has provided 156 training programs serving 18,584 public health professionals through its non-academic training, enrolled 42 workforce members into academic public health programs, and secured 52 student field placements in Medically Underserved Areas.

#### Identified Need: Epidemiologic Competency

Curricular Master of Public Health (MPH) competencies, developed by ASPPH in 2006, serve as a guide for developing quality public health education and training programs (1). Epidemiology-specific competencies were developed by a workgroup of faculty, selected leaders from practitioner organizations, and public health programs. The 10 epidemiology competencies focus on preparing graduates to study the patterns of disease and injury in human populations and the application of epidemiology to the control of health problems (see Table 1). It is expected that upon graduation, graduates with an MPH in epidemiology will be able to apply the competencies in the workforce.

**Table 1:** **Epidemiology core competencies for all Master of Public Health (MPH) graduates**.

Upon graduation, a student with an MPH should be able to
1. Identify key sources of data for epidemiologic purposes
2. Identify the principles and limitations of public health screening programs
3. Describe a public health problem in terms of magnitude, people, time, and place
4. Explain the importance of epidemiology for informing scientific, ethical, economic, and political discussion of health issues
5. Comprehend basic ethical and legal principles pertaining to the collection, maintenance, use, and dissemination of epidemiologic data
6. Apply the basic terminology and definitions of epidemiology
7. Calculate basic epidemiology measures
8. Communicate epidemiologic information to lay and professional audiences
9. Draw appropriate inferences from epidemiologic data
10. Evaluate the strengths and limitations of epidemiologic reports

A set of practice-based competencies were also developed in 2006 by the Council of State and Territorial Epidemiologists (CSTE). The Applied Epidemiology Competencies (AECs), developed by an interdisciplinary workgroup of representatives from federal, state, and local public health agencies, national professional organizations, and schools of public health, span the career ladder from entry level to subject area expert (2, 3). Developed to improve the practice of epidemiology within the public health system, AECs were written for three target audiences: practitioners, employers, and educators. Competencies are defined for four tiers of practicing epidemiologists, based on level of responsibility, experience, and education (3).

**Figure 1:**
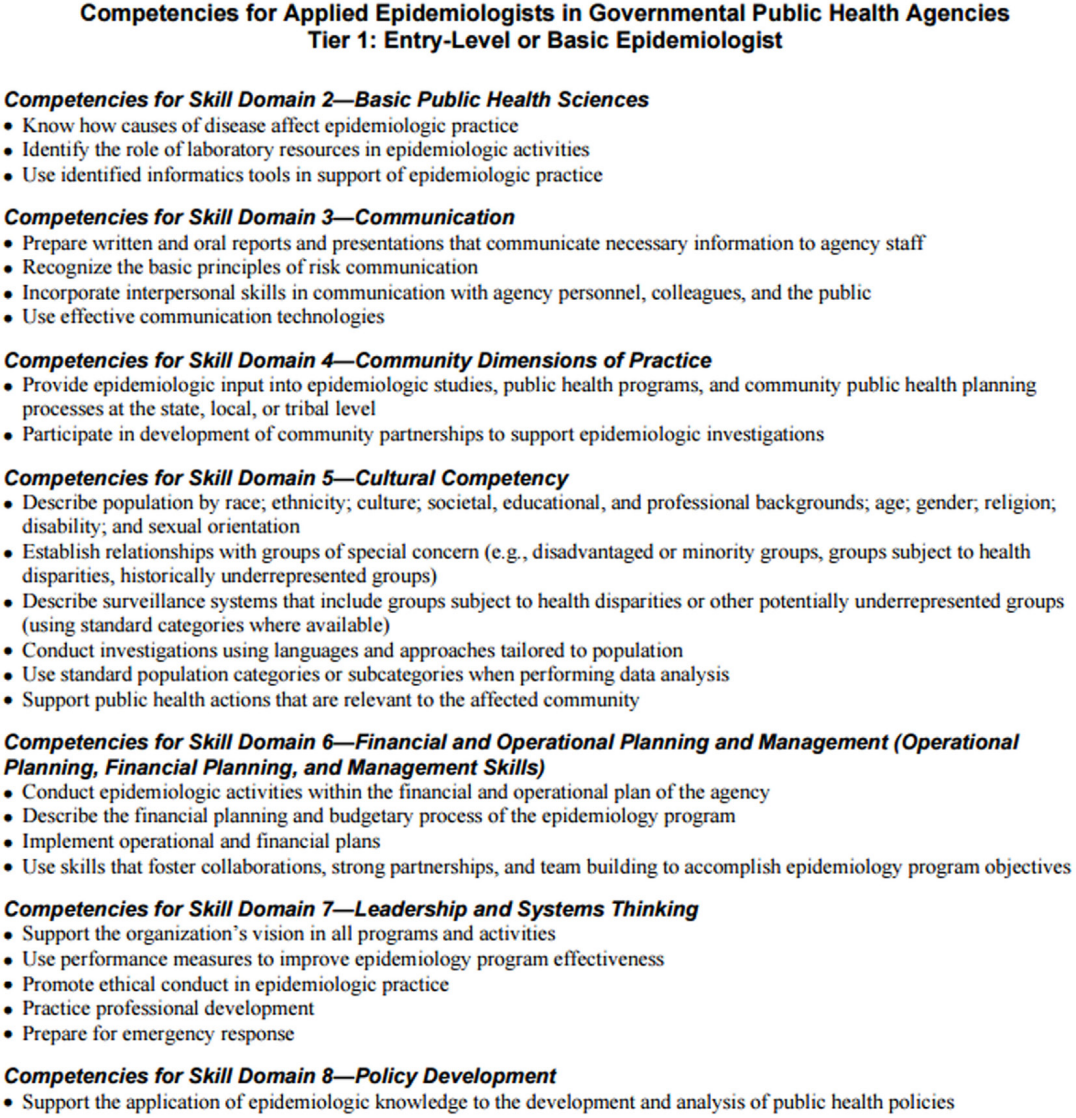
**Tier 1 competencies for applied epidemiologists in governmental public health agencies**.

While both sets of competencies were developed to better align collaboration between academia and practice, implementation of curricular and practice competencies can prove difficult. Ideally, curricular competencies for the MPH graduate would support workforce competencies for tier 1 (entry level) epidemiologists (Figure 1). However, it is pertinent that academic-practice partnerships collaborate to ensure that students are prepared for the workforce in both curricular and practice-based competencies.

#### Identified Need: Workforce Development and Capacity

The need for professional training for the public health workforce has been well documented at the national level. Healthy People 2010 and 2020 recognized that a diverse and prepared public health workforce is a critical underpinning for achieving better health and eliminating health disparities (4). Specific objectives were included pertaining to the public health infrastructure that address continuing education and training for the workforce. In a companion document to Healthy People 2010, the HRSA identified workforce development as the “key ingredient” of the national prevention agenda (5).

The 2003 Institute of Medicine report found that an estimated 80% of the public health workforce had little or no formal professional training in public health or in their specific field within public health (6). For instance, more than 30% of states expressed a need for additional staff training for 23 of 27 applied epidemiology competences, and 43% of states reported minimal to no capacity in research for new insights and solutions to health problems in the epidemiology workforce (7). The Council of State Governments in 2003 surveyed human resources directors in state public health agencies regarding workforce issues. The survey revealed high vacancy rates, high turnover rates, aging of the workforce, and chronic shortages in some occupational classes as major challenges facing public health agencies (8).

The ASPPH reported that 250,000 new public health workers would be needed, nationwide, by 2020 to meet previously established levels (9). It also suggested that as many as 23% of existing public health workers would be eligible to retire by 2012. All states report a need for more epidemiologists, informaticians, and environmental health professionals (10–12). CSTE found that the number of epidemiologists in state health departments decreased by 12% from 2004 to 2009 (13). These statistics, combined with the economic crisis that state and local health agencies are still encountering and the subsequent slow-down in hiring, suggest that it will be increasingly difficult to identify and hire new public health workers, especially in rural and socioeconomically disadvantaged regions, including Kentucky, North Carolina, Tennessee, and Virginia (14–17).

#### Opportunity for Collaboration

In September 2014, Tennessee LIFEPATH won the Public Health Traineeship Program award from HRSA to create the Tennessee Public Health Traineeship (TNPHT). The goal of TNPHT was to recruit and retain students into the Masters of Public Health for three public health discipline shortage areas: Biostatistics, Epidemiology (online and on-ground), and Environmental Health. Through provision of graduate training in the College of Public Health’s MPH program, field placement in Medically Underserved Areas and/or Health Professional Shortage Areas, and field application in one of the College’s Academic Health Departments, students may gain the necessary skills and knowledge to be competitive and successful in the three fields. TNPHT, due to the elimination of federal funding, was scheduled for discontinuation after August 2016. However, 22 MPH students in Biostatistics, Epidemiology, and Environmental Health concentrations were successfully recruited into the program and were on tracks to fulfill the goals of the program by program’s end.

After launch of TNPHT, Tennessee LIFEPATH very quickly began working with TDH to identify and recognize opportunities for training these students in public health practice to more appropriately align theory and application, with special focus in epidemiology as the majority of students enrolled in the program held that concentration. TDH strives to be a trusted and recognized partner in public health, and provides extensive services and coordination of care for residents and visitors of Tennessee. TDH, as the largest public health employer in the state, and through its commitment to a well-trained, competent workforce, showed interest in development of new ways to prepare students. Multiple divisions of TDH hosted interns, fellows, and other trainees over the past 30 years which, in turn, has made TDH a top choice in traineeship locations for public health practice. Therefore, they commenced working with LIFEPATH to establish a new training platform. The intent of the new path was to crosswalk practice and curricular epidemiologic competencies in a way to deliver training through modular, topic focused learning activities. These activities are able to be implemented online or on-ground and fulfill all required learning objectives for the course, but in a manner that builds capacity for practical application. The need for such training was evident by the lack of current, competency-focused training, and the call for innovative mechanisms of delivery to increase the uptake and completion.

The recognized need for trained and competent epidemiologists to enter public health practice spurred the collaboration between the TDH and ETSU. Providing students with classroom-based instruction and assignments that would be similar to those they would be expected to complete in an entry-level epidemiologist position could help guide students toward public health practice post-graduation and prepare them for the rigors of those positions. This high need for well-trained, competent epidemiology personnel who are well suited to expeditiously apply their gained skill set directly after obtainment of their degree and assurance that their academic training contained real-world application opportunities was the goal of this collaboration and training.

## Methods

### Objectives and Approach

The MPH coursework can be completed either in person or online. This degree program was designed to enable working professionals and other students requiring flexibility in learning to attain an advanced degree in public health. The epidemiology concentration requires nine credit hours in epidemiology electives in addition to a set of core MPH and epidemiology concentration requirements. To complete the program, each student must also complete a field experience (6 credit hours and 300 contact hours) in public health.

Tennessee Department of Health, located in Nashville, TN, USA, is focused on protecting, promoting, and improving the health of all of those who live in and visit Tennessee. A part of that mission is to educate and train public health professionals who will be trusted partners across agencies and the state. ETSU approached TDH with the idea for a partnership to build three asynchronous epidemiology modules to be delivered online to an interdisciplinary audience of graduate students. This was an optimal partnership due to the previous integration of students into TDH and the ability for TDH to provide a public health practice perspective to a wider group of students. Topic-specific modules were created that would be used within the concentration epidemiology courses on the topics of infectious disease and chronic disease, and the elective course on the topic of cancer epidemiology.

These modules were piloted during the 2015–2016 academic term. The infectious disease epidemiology module was delivered in the fall 2015 semester, while the chronic disease and cancer epidemiology modules were to be administered in 2016. The ETSU/TDH team conducted regular interactions for feedback on how the modules were performing with the students and to adjust any parts of the modules, which may have been unsuccessful. Additionally, the instructor for the infectious disease course was a member of the ETSU/TDH team to develop the modules so when a change was necessary, the change was made by emailing the TDH staff and discussing the change as well as the rationale behind it. The team would then discuss and determine an alternate plan.

### Process

In the spring of 2015, the ETSU/TDH team began having conference calls between the two institutions. In the first call, the format, intentions, and goals of the modules were discussed. The goals of the modules were to (1) introduce students to a public health issue, (2) provide students with hands-on learning about data and information available through TDH, and (3) allow students to connect theory to practice by having them create a product for use by TDH. ETSU requested modules for infectious disease, chronic disease, and cancer epidemiology be completed in August 2015 for fall 2015, spring 2016 piloting to students. The infectious disease epidemiology would be the only module piloted in the fall due to the course schedule.

The modules were created separately using three different methods. The infectious disease module was based on classroom case studies produced by the U.S. Department of Health and Human Services, Public Health Service, Centers for Disease Control and Prevention (http://www.cdc.gov/epicasestudies/). These case studies have both a student and instructor version, which provides guidance as to how the case study should be implemented. For the purposes of the ETSU/TDH modules, TDH selected the “Cryptosporidiosis in Georgia” case study (see Figure 1 for CDC case study objectives). Briefly, this module was designed to teach students about water-borne diseases, and how outbreaks are recognized and evaluated for mitigation and prevention. After completion of the CDC case study section of the module, students were asked to navigate the TDH Communicable and Environmental Disease and Emergency Preparedness (CEDEP) dashboard for cryptosporidiosis, answer questions about the disease burden in Tennessee during 2013, and then create fact sheets for two public audiences to communicate risk factors and preventative measures. Students chose two of the following: day-care centers, hospitals, farmer’s markets, or recreational water parks/pools. The modules included informational links regarding writing materials that met health literacy guidelines for the public audience. Students were required to submit the fact sheets for grading by ETSU faculty as well as a representative from TDH. This module was piloted in fall 2015 through the infectious disease epidemiology course. Thirty-two students participated and submitted fact sheets to ETSU faculty and the TDH representative. Students were predominately MPH epidemiology students (*n* = 19), with 10 onsite students and 9 online. Other students included MPH students with other concentrations (*n* = 5, all onsite), graduate certificate in epidemiology students (*n* = 3, all online), DrPH epidemiology students (*n* = 2, all onsite), and dual degree students: MD/MPH (*n* = 2, all online) and PharmD/MPH (*n* = 1, onsite).

The chronic disease epidemiology module was constructed entirely using data and questions originating at TDH. The topic of this module was “Childhood Asthma in Tennessee Emergency Rooms.” In accordance with the TDH focus on reducing tobacco use in Tennessee, childhood asthma rates are related by its association with secondhand tobacco smoke. The module features data from the Tennessee Hospital Discharge Data System (TN HDDS) 2013 data year. TN HDDS is an administrative billing system used to capture a variety of diagnoses and procedures that occur in Tennessee hospitals. Students were provided the TN HDDS manual as well as a systematic review of asthma case ascertainment in claims data. Following this section and additional reading selections, questions were included to review the content.

The students are prompted within the written module to access literature review webinar slides which they could review at their pace. The slides were intended to assist students in searching and synthesizing the literature. Students are tasked with searching the literature for a number of selected peer-reviewed manuscripts in PubMed. Subsequent questions were asked regarding Medical Subject Heading (MeSH) terms and their use in the literature review process. The chronic disease epidemiology module contained a focused section on problem statement development. This piece of the module was to be performed as homework after the in-class lesson on problem statements. The assignment in this section was to have problem statements for childhood asthma in Tennessee based on a literature review to be conducted by the students and the previously provided reports.

Finally, data analysis and interpretation are covered in the last piece of the module. Using data provided by TDH, students must identify the counties and region with the highest rate of childhood asthma emergency room visits and use TDH websites to explore pertinent characteristics of these geographical locations. As with the infectious disease epidemiology module, the final project for this module will be fact sheets about childhood asthma for one of the counties with the highest rates of childhood asthma emergency room visits. The fact sheets should include a background of the problem, a description of the risk factors, graphical and written representations of the problem or distributions associated with childhood asthma, and conclude with suggestions for evidence-based interventions to reduce childhood asthma in that population.

The infectious disease and chronic disease epidemiology modules were presented to ETSU staff for critique and edits at an in-person meeting during the summer. The cancer epidemiology module could not be created to its full extent prior to the start of the course. This module will be added for the 2016–2017 school year. The ETSU/TDH team reviewed the modules for pace, context, content, and additions/corrections. TDH staff were able to ask questions regarding the availability of resources, such as SAS software and digital libraries. ETSU staff assured TDH that resources were available to students to allow them to complete the modules successfully. Much of the feedback TDH received from ETSU centered around the method of delivery. TDH had not specified how each section was intended to be delivered to the students. The ETSU/TDH team decided to present the pilot module, infectious disease, to students at the end of the course.

During this meeting, it was also determined that the product to be created by the students would be fact sheets or reports on the disease presented in the module. The ETSU/TDH staff also agreed that this would be a worthwhile exercise for students to learn to synthesize scientific information, create esthetically pleasing products, and adjust language to meet health literacy standards. The methods of delivery for each section within a module varied from recorded webinar format to self-guided instruction. One module utilized available learning tools provided by CDC, while the other module was constructed entirely using TDH data. Both modules included various exercises and assignments to be conducted in class and as homework, and concluded with the student being asked to construct a learning product as a final project. The ETSU–TDH team decided that this learning product would be provided back to TDH for possible future use.

### Evaluation

The infectious disease module was the pilot module to inform the process, and the student outcomes associated with the course. Modules were graded quantitatively for their course grade (academic evaluation), and the fact sheets received comments from a TDH staff member *via* a qualitative rubric for end-user utility (practical evaluation). The two rubrics enhanced product development by capitalizing on the unique perspectives of the ETSU faculty and the TDH staff. Faculty members often have regular interaction with students and are familiar with the level of work they are capable of producing. In turn, TDH staff were not familiar with the students and could evaluate the students’ work objectively. Development of shared metrics of student evaluation, like the two rubrics, decreases the learning curve once employed and prepares students for critical feedback.

**Figure 2:**
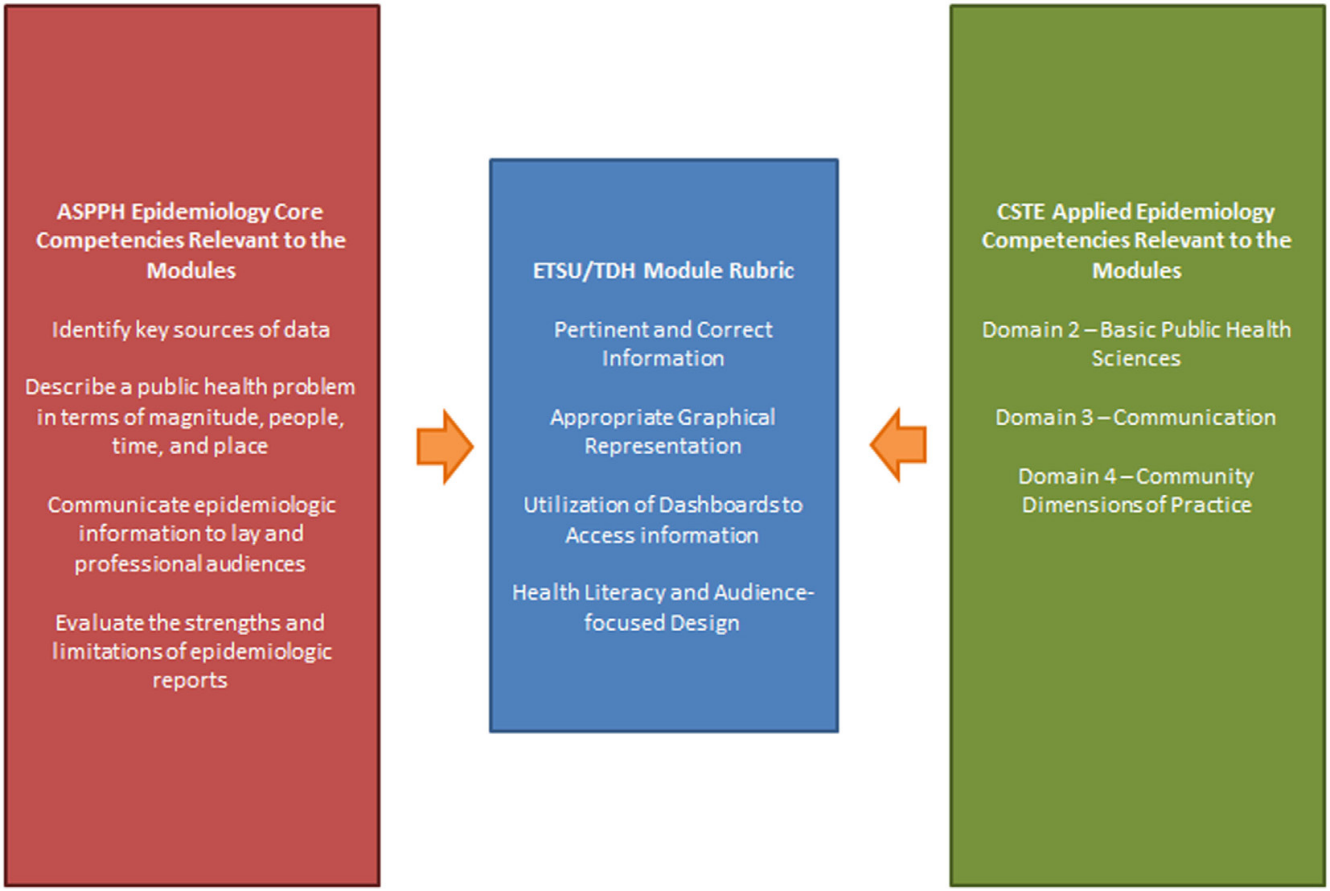
**The integration of the academic and practical competencies to create the ETSU/TDH module rubric**.

The academic evaluation for the infectious disease modules scored the students’ module participation and the completion of assigned exercises. The fact sheets were graded for usefulness and accuracy of information, as well as accessibility when presented to the public. Students could receive 25 points for this exercise and scores ranged from 18 to 25. The exercise was included in “exercises” portion of the student’s grade (20% of overall grade, four exercises in the course). The academic and practical rubrics closely mirrored one another and were informed by the ASPPH Epidemiology and AECs (see Table 2; Figure 2). The academic rubric provided scores for each of the assessed criteria.

**Table 2:** **Academic and practical evaluation rubrics for the infectious disease module**.

Academic criteria	Points	Practical criteria	Priority
Submitted on time	2.5		N/A
CEDEP dashboard exercise	5		N/A
Is the information useful and factual? • Does the fact sheet give additional pertinent facts about the disease that someone who has never heard of it might find useful? • Does the fact sheet contain information on where and how to find out more about this disease? • Is there anything on the fact sheet that is not accurate?	8	Organization and information • Does the fact sheet contain a description of the disease, transmission, symptoms, and treatment and prevention facts? • Does the organization of the fact sheet make sense visually? • Is the information contained in the fact sheet accurate?	1
Is the information accessible to the general public? • Is the health literacy level appropriate? • Are jargon and discipline-specific language replaced with language appropriate to a lay audience?	7	Target Audience Tailoring • Is the health literacy level above or below what is appropriate for the audience? • Does the fact sheet apply to or appeal to the stated target audience?	3
Are graphics or illustrations used effectively?	2.5	Graphics • Are the graphics relevant to the subject matter? • Do the graphics support the public health message? • Are the graphics appropriate for the audience? • Do the colors maximize visibility and readability?	2
Total	25		N/A

Since the TDH staff were not providing a grade, *per se*, to the students, the comments were prioritized from 1 to 3 in order of importance. For example, if a fact sheet’s information was accurate and the graphical representations were optimal while the literacy level may have been too high for the target population, the student received highly favorable comments. However, students who presented information in outline format without graphics or consideration for the target audience, the comments they received were less favorable. A number of students (less than 1/3) created outlines of the information rather than fact sheets. This demonstrated a need for the students to have additional instruction within the module or within the coursework as to the components of fact sheets and how fact sheets are best utilized. After the academic and practical evaluations, several students were selected to have their fact sheets reviewed by senior epidemiologists in TDH’s CEDEP division. Examples of feedback provided to students are listed below:

TDH staff

- While the facts are great and the recommendations pertinent, if you saw this hanging up on a wall or in a physician’s waiting room, would you gravitate toward it?
- Fact sheets are well organized but not really good for the public. Graphs, pictures, etc. would improve this fact sheet.
- Nice job – I would use “crypto” for the majority of the fact sheet for the benefit of health literacy.
- VERY NICE. This fact sheet is approachable and creative. Great use of the risk factors and recommendations to the public. It is extremely clear who your audience is and what this fact sheet is seeking to achieve. Bravo.

ETSU faculty

- Signs and symptoms could be presented as bullets.
- Provide graphics, illustrations, etc. instead of an outline of the crypto.
- Clear fact sheets that provide good details.
- Well done and caters to those with limited health literacy.

Additionally, the academic and practical evaluation added value for the students. The students being unaware of the TDH staff’s evaluation of their fact sheets enabled ETSU faculty to view what students would produce without that knowledge of a third-party observing. Additionally, after the students received the comments from TDH, the implied message was for their future work to be portfolio ready since the document may be shared widely. If the students submit portfolio-ready work, the product can be used in job interviews to display competencies. Students remarked that it was helpful to have their product reviewed externally and expressed an appreciation to have insights about the type of product that TDH was looking for. Further, while students were not asked directly about this module in their student assessment of instruction at the end of the course, the majority of the students in the online and onsite sections agreed that the course helped to increase their knowledge and skills in the content area, and that the course provided meaningful feedback. Further, students had the opportunity to comment on the course and comments included, “the class did a great job not only teaching theory but helping students learn to apply it” and “having TDH involved provided additional real-life examples.”

## Discussion

This partnership between ETSU and TDH marked another groundbreaking milestone in collaboration between state public health agencies and academic institutions. While TDH staff are often invited to lecture, the development of practical modules with relevant and useful end products is a new frontier for both parties. ETSU students were able to utilize real-world data and were afforded the unique perspective and objective feedback of those currently practicing public health. TDH staff gained insight into how potential employees are being trained and provide valuable input to the content construction of that training. The ETSU/TDH team worked closely together to not only formulate the modules but also to improve them during the process. The piloting of one module, instead of all three modules in one semester, allowed for modifications to be made to the modules themselves or the evaluation plan. Additionally, the process was consistently documented to enable this monitoring and to evaluate the process thoroughly. Given, the process was limited by the timeframe from spring to fall; the modules had to be completed quickly. Even so, the modules were thorough and provided students an extensive overview of the topics.

The initiation and implementation of this process is closely aligned with Himmelman’s levels of collaboration (18). The framework describes networking, coordinating, cooperating, and collaborating – with collaborating being the highest level. Prior to the development of the modules, ETSU and TDH often worked together on a cooperating basis, which is defined by Himmelman as “exchanging information for mutual benefit and altering activities and sharing resources to achieve a common purpose.” The institution of these modules moves this partnership up a level to collaboration where we are “exchanging information for mutual benefit and altering activities, sharing resources, and enhancing the capacity of another to achieve a common purpose.” ETSU is, in theory, enhancing the potential workforce capacity and epidemiologic capacity of TDH by providing students with practical experiences and lessons that will serve to increase their value as an employee. One of the key pieces of collaboration is sharing in not only the rewards but also the risks. TDH and ETSU have co-branded the modules to highlight the investments of both parties to make the module process functional.

The development and implementation of these modules demonstrates that the accomplishment of the competencies is now obtained through theory application. The team used academic and practical competencies, both created by national bodies of experts and stakeholders, to inform the rubric by which the modules were graded. Identifying and applying the common competencies between academic public health and public health practice helped us to notice where the public health workforce can become better educated to benefit either aspect of public health. The most recent CSTE Epidemiologic Capacity Assessment (ECA) Report’s Findings and Recommendations identified that a large percentage of states had minimal to no capacity to carry out several Essential Public Health Services (EPHS), and more than 30% of entry- and mid-level epidemiologists reported the need to additional workforce development and training (19). One of the recommendations made was for the review and development of recruitment and retention strategies of epidemiologists in the public health workforce (20). The ETSU/TDH collaboration addresses the concerns raised in the 2013 ECA by providing practice-based training to the future public health workforce while they are still involved in their academic programs. Additionally, familiarizing students with TDH during their academic careers prepares them for interactions and opportunities with state public health to aid in recruitment.

## Limitations

While process measures and immediate outcomes were met with the successful completion of implementation of modules, student participation numbers, and completion of student evaluations, the project is too much in its infancy to speak to the intermediate or long-term outcomes. This collaboration will require further replication and implementation to speak to the external validity. The project can confidently recommend the employed model of collaboration to others with interest in aligning public health academic competencies with those of practice.

## Conclusion

Other state public health agencies and academic institutions could improve on our model of collaboration in ways mostly related to the formative aspects of the process. Teams should be identified, and the process mapped out early on in the timeline. The ability to pilot at least one module proved essential to the success of this collaboration as we were able to assess our efforts almost immediately. Other state public health agencies may wish to integrate their staff into the module administration through having some pieces of the modules be taught to the students by staff. Depending on workforce needs and the current capacity, agencies may wish to construct multiple modules surrounding different topics and solicit products from students that may be used publicly. This would enhance the shared benefit of the modules and strengthen the portfolios and exposure of the students.

As public health evolves to spanning academia and the practical application of the competencies, it will become imperative for the workforce to be skilled in both aspects of the field. This innovative and imperative partnership between a state public health agency and an academic institution unveiled a necessary opportunity for such collaborations. Not only did this provide an excellent opportunity for student learning and training but also provided greater insight into academia and public health practice, and strengthened the depth of the partnership between ETSU and TDH. Students were provided with a unique opportunity for hands-on practical training and provided a deliverable that could be used in the workforce. Academia and practice gained additional information on the types of training needed, delivery methods, instructions, and the deliverables. Finally, moving the level of collaboration from cooperating to collaboration along Himmelman’s scale illustrates the depth of the academic-practice partnership and potential for future projects.

## Author Contributions

KG, PM, and MQ contributed to the idea and framework of the manuscript, drafted several parts of the manuscript, and provided edits and final content.

## Conflict of Interest Statement

The authors declare that the research was conducted in the absence of any commercial or financial relationships that could be construed as a potential conflict of interest.
